# Failure of SOX9 Regulation in 46XY Disorders of Sex Development with SRY, SOX9 and SF1 Mutations

**DOI:** 10.1371/journal.pone.0017751

**Published:** 2011-03-11

**Authors:** Kevin C. Knower, Sabine Kelly, Louisa M. Ludbrook, Stefan Bagheri-Fam, Helena Sim, Pascal Bernard, Ryohei Sekido, Robin Lovell-Badge, Vincent R. Harley

**Affiliations:** 1 Molecular Genetics and Development, Prince Henry's Institute, Melbourne, Victoria, Australia; 2 Department of Anatomy and Developmental Biology, Monash University, Clayton, Melbourne, Australia; 3 Department of Molecular Biology and Biochemistry, Monash University, Clayton, Melbourne, Australia; 4 Division of Developmental Genetics, MRC National Institute for Medical Research, The Ridgeway, Mill Hill, London, United Kingdom; University of Illinois at Chicago, United States of America

## Abstract

**Background:**

In human embryogenesis, loss of *SRY* (sex determining region on Y), *SOX9* (SRY-related HMG box 9) or *SF1* (steroidogenic factor 1) function causes disorders of sex development (DSD). A defining event of vertebrate sex determination is male-specific upregulation and maintenance of *SOX9* expression in gonadal pre-Sertoli cells, which is preceded by transient *SRY* expression in mammals. In mice, *Sox9* regulation is under the transcriptional control of SRY, SF1 and SOX9 via a conserved testis-specific enhancer of *Sox9* (*TES*). Regulation of *SOX9* in human sex determination is however poorly understood.

**Methodology/Principal Findings:**

We show that a human embryonal carcinoma cell line (NT2/D1) can model events in presumptive Sertoli cells that initiate human sex determination. SRY associates with transcriptionally active chromatin in NT2/D1 cells and over-expression increases endogenous *SOX9* expression. SRY and SF1 co-operate to activate the human *SOX9* homologous TES (*hTES*), a process dependent on phosphorylated SF1. SOX9 also activates *hTES*, augmented by SF1, suggesting a mechanism for maintenance of *SOX9* expression by auto-regulation. Analysis of mutant SRY, SF1 and SOX9 proteins encoded by thirteen separate 46,XY DSD gonadal dysgenesis individuals reveals a reduced ability to activate *hTES*.

**Conclusions/Significance:**

We demonstrate how three human sex-determining factors are likely to function during gonadal development around *SOX9* as a hub gene, with different genetic causes of 46,XY DSD due a common failure to upregulate *SOX9* transcription.

## Introduction

DSDs are among the most common genetic diseases in humans referring to a group of congenital conditions in which the development of the chromosomal, gonadal or anatomical sex has been abnormal [1]. Mutations in the key testis-determining factor *SRY* result in 46,XY DSD. Significantly, almost all 46,XY female patients with *SRY* mutations show complete gonadal dysgenesis [2], [3], consistent with the function of *SRY* acting early in the development of the embryonic testis. The incidence of *SRY* mutations in 46,XY DSD is however quite small (10–15%) and does support the notion that genes other than *SRY* are essential for proper testis development. Despite the ongoing identification of a number of these key testis-determining genes [4], most of which are transcription factors, the actions, co-factors and downstream targets of human SRY have proven difficult to ascertain.

*SRY* which is expressed in Sertoli cells plays key cellular roles in the developing gonad including the differentiation of Sertoli cells [5]; inducing migration of cells from the mesonephros into the gonad [6]; inducing proliferation of cells within the gonad [7]; inducing the development of the vasculature patterning of the XY gonad [8]; and glycogen accumulation in pre-Sertoli cells [9]. Each role may be mediated by a direct interaction between SRY and one or more partner proteins on one or more independent target genes. Hence, one question arising is whether the various and multiple roles played by SRY are direct or indirect?

The human *SOX9* gene when mutated causes CD/SRA1 (Campomelic Dysplasia/Autosomal Sex Reversal), and has become known as a pivotal sex-determining gene [10], [11]. The upregulation, sexual dimorphic expression pattern and conserved protein structure of SOX9 are consistent across all vertebrate species, regardless of the switch mechanism controlling sex determination, being SRY in mammals (except for the mole vole, [12]), ZW chromosome gene(s) in birds [13] and temperature sensitivity of egg incubation in turtles and crocodiles [14], [15]. In XX *Sox9* transgenic mice, and probably also in human XX males with *SOX9* duplications or translocations, the increased levels of SOX9 are sufficient to initiate testis formation in the absence of *Sry/SRY* [16], [17], [18], [19]. This raised the possibility that *Sox9/SOX9* might be a direct and potentially only target for SRY. In agreement, the recent identification of a conserved testis-specific enhancer of *Sox9* (*TES*) in the mouse has revealed a co-transcriptional network of SRY, SF1, and SOX9 involved the direct initiation, upregulation and maintenance of *Sox9* expression in the mouse XY gonad [20]. Like *SRY* and *SOX9*, mutations in human *SF1* lead to 46,XY gonadal dysgenesis [21], [22], [23].

While in the mouse, SRY directly up-regulates *Sox9* expression to induce testis development [20], the relationship between human SRY and *SOX9* is less clear. The understanding of human SRY protein function is hampered by its lack of protein sequence conservation across mammalian species. Protein structural domains of SRY are poorly conserved, the only conserved domain between human and mouse is the high mobility group (HMG) domain [24], yet human SRY under the control of mouse regulatory sequences can still induce testis development in XX transgenic mice [25]. The importance of the HMG domain in the function of the human SRY protein is also highlighted by the fact that most 46,XY gonadal dysgenesis mutations cluster within this domain. It has thus been proposed that human SRY instigates testis-determination by potentially (i) activating gene expression through its consensus binding site (A/T)AACAAT [26], (ii) functioning as an architectural factor by bending DNA [27], (iii) repressing a putative suppressor of a testis-promoting factor [28], and (iv) being involved in pre-mRNA splicing [29]. The lack of a suitable *in vitro* model system, including a bone fide SRY testis-determining target gene, has hindered the ability to test such hypotheses of human SRY.

In the current study, we aimed to develop an *in vitro* assay to understand the molecular actions of human SRY in sex determination. We demonstrate that endogenous *SOX9* is upregulated in the human embryonal carcinoma cell line NT2/D1 over-expressing SRY, a model of presumptive Sertoli cells [30]. This upregulation is associated with SRY localisation to actively transcribed chromatin and not pre-mRNA splicing complexes. Furthermore, we reveal that the human homologous *SOX9* testis-specific enhancer (*hTES*) is responsive to human SRY, SF1 and SOX9 co-transcriptional activation. However, mutant SRY, SF1 and SOX9 proteins encoded by 46,XY DSD individuals exhibit a reduced ability to activate *hTES*. As a central hub gene, *SOX9* regulation is an important event in mammalian sex determination. This study provides an important insight into the molecular actions of human SRY in this process. Furthermore, by assaying SRY, SF1 and SOX9 from 46,XY DSD individuals we have provided functional evidence of mutations that result in reduced SOX9 expression.

## Results

### SRY up-regulates *SOX9*/SOX9 expression in the human NT2/D1 cell line

**Figure 1:**
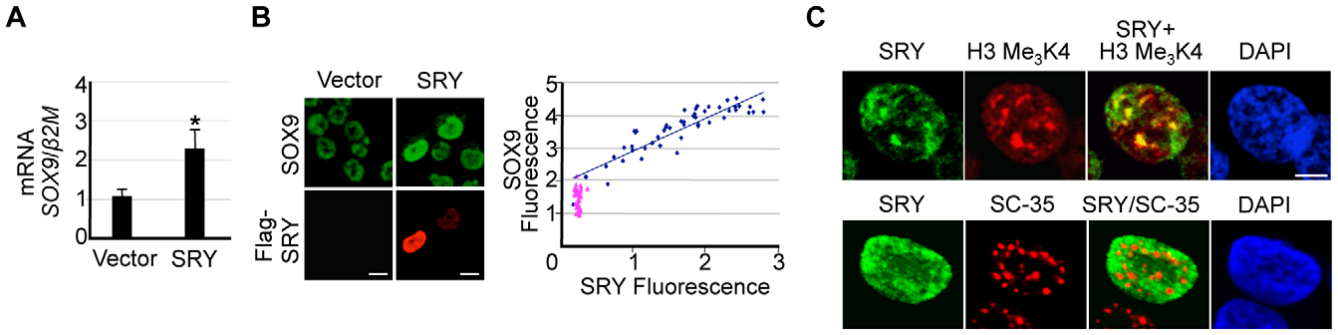
Endogenous *SOX9* is up-regulated in NT2/D1 cells in response to SRY. (**A**) *SOX9* is significantly upregulated in NT2/D1 cells transiently overexpressing SRY (1 µg), as measured by QRT-PCR and compared to empty vector (*n* = 3). (**B**) Immunostaining for endogenous SOX9 and exogenous SRY (Anti-Flag) reveals a positive correlation between SOX9 fluorescence and exogenous SRY fluorescence (R^2^ = 0.835, *n* = 50). Scale bar represents 10 µm. Each point averages three fluorescence readings per NT2/D1 cell (Blue Diamond - SRY-transfected cells; Pink triangle - non-Flag cells). (**C**) (Upper panel) H3 Me_3_K4 (red) is exclusively nuclear in NT2/D1 cells. Overlap of SRY (green) and H3 Me_3_K4 in NT2/D1 cells reveals co-localization (yellow) within nuclear compartments. (Lower panel) SC-35 (red) is localized to alternate nuclear compartments, as overlap images of Flag-SRY and SC-35 staining do not show co-localization. DAPI (blue) stains nuclear DNA. Scale bar represents 10 µm. Error bars represent the standard error of mean values. Two-tail t-Test of paired sample means was performed. * *P*<0.05.

To test whether human SRY can activate *SOX9* transcription we evaluated the human embryonal carcinoma cell line, NT2/D1, as a model of presumptive Sertoli cells. NT2/D1 cells express many of the genes implicated in testis differentiation, including *SRY*, *SOX9* and *SF1* [30]. To simulate the onset of SRY expression, we transiently transfected *SRY* into NT2/D1 cells. A significant 2.2-fold increase in *SOX9* mRNA was measured by quantitative RT-PCR (QRT-PCR) (Fig. 1A). SOX9 protein levels increased accordingly, as represented by the positive correlation between exogenous SRY and endogenous SOX9 immunofluorescence (Fig. 1B). *In vitro*, SRY can act as an architectural transcription factor, binding and bending specific DNA sequences or structures [31]. We co-immunostained NT2/D1 cells to detect both SRY and tri-methylated histone 3 lysine 4 (H3 Me_3_K4), which is exclusively associated with actively transcribed chromatin regions [32]. The co-localization of SRY and H3 Me_3_K4 proteins within the nucleus of NT2/D1 cells suggests that, when upregulating *SOX9*, SRY functions in a transcriptional complex (Fig. 1C, upper panel). It has been proposed that SRY also acts in pre-mRNA processing [29], however, in our study, and in agreement with others [33], Flag-SRY protein does not co-localize with splicing factor SC-35 (Fig. 1C, lower panel).

### SRY, SF1 and SOX9 activate the human homologue of the testis-specific enhancer of *Sox9 (hTES)*

**Figure 2:**
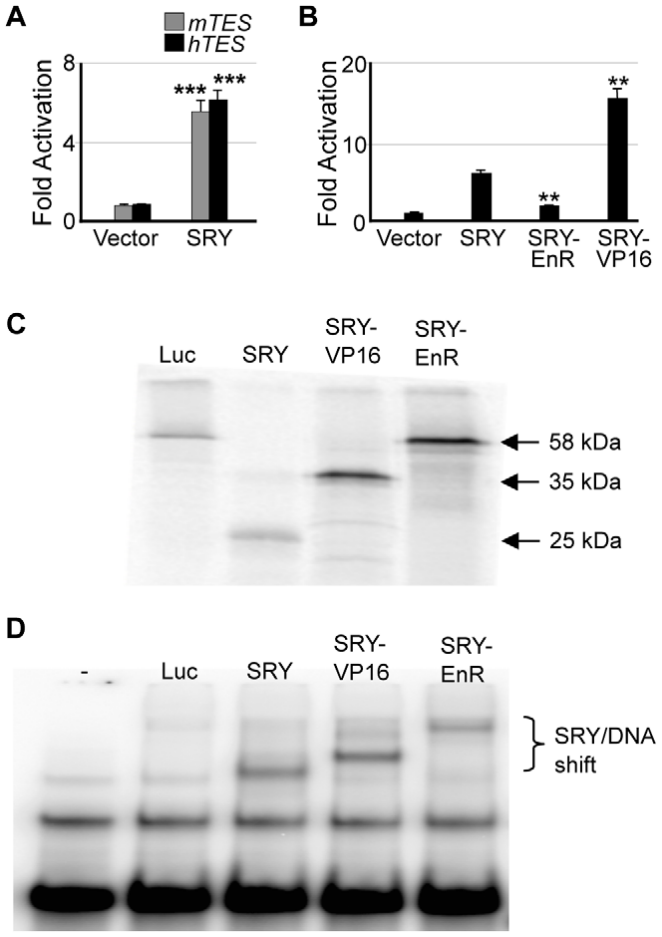
SRY acts as a transcriptional activator to stimulate the human homologous testis-specific *SOX9* enhancer (*hTES*). (**A**) *mTES* and *hTES* reporter constructs (1.6 µg) are activated ∼6-fold in CHO cells by exogenous human SRY (100 ng) compared to vector alone. (*n* = 3–5) (**B**) SRY fusion to VP16 (SRY-VP16, 104 ng) increased activation of *hTES*, whereas SRY fusion to EnR (SRY-EnR, 120 ng) reduced activation compared to SRY (*n* = 2). All reporter assays conducted in duplicate. Error bars represent the standard error of mean values. Two-tail t-Test of paired sample means was performed. *** *P*<0.001, ** *P*<0.01. (**C**) *In vitro* translated SRY-VP16 and SRY-EnR fusion proteins migrate at their expected product size compared to SRY WT. (**D**) Electrophoretic mobility shift assay demonstrates that *in vitro* translated SRY-VP16 and SRY-EnR fusion proteins bind to a ^32^P radio-labelled DNA oligonucleotide containing the SOX DNA binding consensus sequence AACAAT. SRY WT also binds this oligonucleotide whereas the *luciferase* control protein cannot.

SRY/SOX9 and SF1 synergistically activate a ∼3.2 kb, testis-specific enhancer of the mouse *Sox9* gene, 13.2–10.1 kb upstream of the transcription start site (termed *mTES*) [20]. *mTES* is highly conserved across species including human [20], [34]. What we believe to be the equivalent human enhancer element (termed *hTES*) is positioned 14.7–11.6 kb upstream of the transcriptional start site of the human *SOX9* gene. To test whether SRY could activate the human enhancer, *hTES* and *mTES* (as control) sequences were cloned into a reporter construct and each was co-transfected with a human SRY expression vector into Chinese Hamster Ovarian (CHO) cells that lack endogenous Sry but confer SRY transcriptional activity [35]. We also obtained similar results using HEK293 cells, derived from a human embryonic kidney (results not shown). Both enhancers were stimulated by human SRY to a similar extent (Fig. 2A), whereas SRY did not activate the empty E1b-luc reporter vector (data not shown). This indicates that SRY-mediated activity was via the human and mouse *SOX9/Sox9* enhancer sequences. Within the transcriptional complex, SRY has been proposed to function either as a transcriptional activator [26] or as a repressor of a transcriptional repressor [28]. We fused SRY to the activation domain of the viral protein VP16 [36] and to the repressor domain of the *Drosophila melanogaster* protein Engrailed (EnR) [37]. Both SRY fusion proteins were stably translated and bound SRY consensus DNA sequences with wild-type affinities (Fig. 2C and 2D respectively). If SRY were a repressor of a repressor of SOX9, SRY-VP16 would decrease, whereas SRY-EnR would increase *hTES* activity in comparison to wild-type SRY. However, fusion to VP16 significantly increased SRY-mediated *hTES* activation 2-fold, whereas SRY-EnR decreased *hTES* activation 3-fold (Fig. 2B). Taken together, these data support a model where SRY functions as a transcriptional activator.

**Figure 3:**
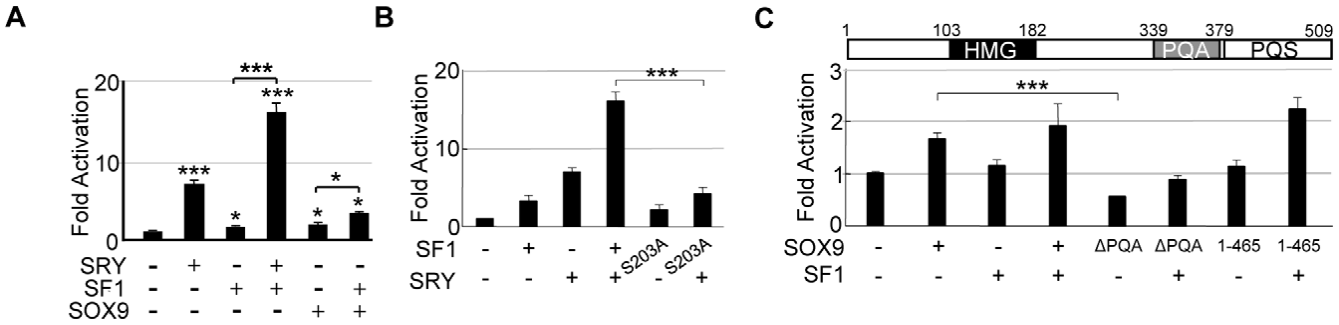
Co-operative regulation of the *SOX9* enhancer by SRY, SF1 and SOX9. (**A**) Transfection of CHO cells with exogenous SF1 (20 ng) stimulates *hTES* ∼1.8-fold compared to vector. Together, SRY and SF1 co-operate to activate *hTES* by ∼16-fold. SOX9 (26 ng) activates *hTES* ∼2-fold compared to vector, increased to ∼3-fold by the presence of SF1. (**B**) Transfection of S203A SF1 phosphorylation mutant (100 ng) had similar transcriptional activity of the *hTES* reporter construct in comparison to SF1 WT (100 ng). Co-operative effect between SRY and SF1-S203A had abolished *hTES* reporter activity in comparison to SRY and SF1 WT co-transfected cells. (**C**) SOX9 truncation protein (amino acids 1–465) lacking the PQS transcriptional activation domain did not activate the *hTES* reporter construct to the same extent as SOX9 WT either alone or in the presence of SF1. Removal of the PQA transcriptional activation domain did not have any effect on activation of the *hTES* reporter construct compared to SOX9 WT. All experiments conducted three times in duplicate. Error bars represent the standard error of mean values. Two-tail t-Test of paired sample means was performed. * *P*<0.05, *** *P*<0.001.

We next tested the effects of SF1 on SRY-mediated *hTES* activity. Transfection of exogenous SF1 into CHO cells, which contain low levels of endogenous SF1 [22], activated the *hTES* enhancer 1.8-fold, while SRY activated *hTES* 8-fold. Together, SRY and SF1 stimulate *hTES* activity 16-fold, suggesting co-operative regulation of the *SOX9* enhancer is occurring (Fig. 3A). Phosphorylation of SF1 appears to be essential for this co-operation with SRY, as co-operativity was abolished when the SF1-S203A phosphorylation mutant was used (Fig. 3B). This mutant is known to disrupt co-factor recruitment and, as a consequence, SF1 transcriptional activity [38].

Previously, it has been shown that in the presence SF1, SOX9 can synergistically activate *mTES* [20]. To investigate whether SOX9 could help maintain its own expression also on the human enhancer, a SOX9 expression plasmid was co-transfected with the *hTES* reporter construct. SOX9 significantly stimulated *hTES* reporter activity 2-fold (Fig. 3A). In the presence of SF1, SOX9 mediated stimulation increased to 3-fold. SOX9 mutations causing 46,XY gonadal dysgenesis frequently truncate the C-terminus leading to loss of PQS and PQA transactivation domains [2]. Deletion analysis indicated that the PQS transactivation domain was essential for the *hTES* enhancer activation that we observed, whereas the PQA domain was not (Fig. 3C). The data suggest that DNA binding and the PQS-region, but not dimerization, are required for normal SOX9 activity.

Taken together, these data support a model of human gonadal development whereby SF1 participates firstly with SRY to up-regulate *SOX9* expression, and then with SRY, SF1 and SOX9 to maintain its own expression. These findings agree with data obtained with *mTES* in COS cells [20], although here we observe a co-operativity rather than a synergy between SF1 and SOX9.

### SRY, SF1 and SOX9 mutant proteins fail to activate the *hTES* enhancer

**Figure 4:**
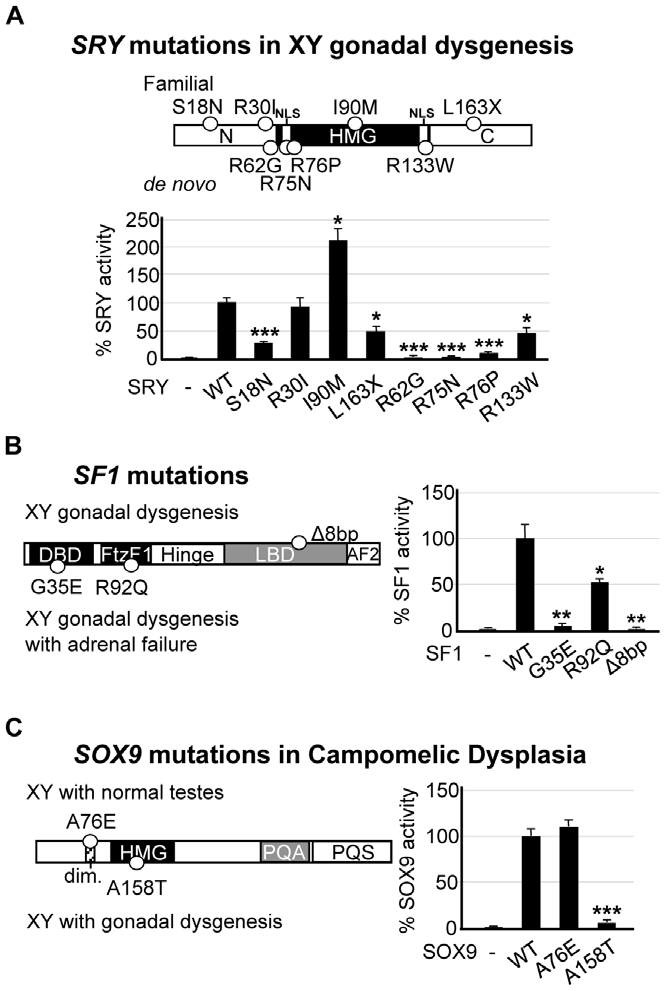
Relating *SOX9* gonad-specific enhancer activity to disorders of sexual development caused by mutations to SRY, SF1 and SOX9. (**A**) Locations of four *de novo* and four familial SRY mutations causing 46,XY gonadal dysgenesis (Table 1). CHO cells were co-transfected with *hTES* reporter and constructs expressing the SRY mutants (100 ng). Transcriptional activity was plotted as a percentage of SRY wild-type (WT) and statistical analysis performed. HMG, high mobility group domain. (**B**) Locations of two SF1 mutants causing 46,XY gonadal dysgenesis and adrenal failure (G35E and R92Q) and one mutant causing 46,XY gonadal dysgenesis (Δ8bp) (Table 2). All SF1 mutants (20 ng) showed reduced transcriptional activity, plotted as a percentage of SF1 WT and statistically compared. DBD, DNA binding domain; FtzF1, FtzF1-box (A-box); LBD, ligand binding domain; AF2, activation function-2. (**C**) 46,XY gonadal dysgenesis mutant SOX9-A158T (26 ng) failed to activate *hTES* compared to SOX9 WT (26 ng), whereas 46,XY mutant SOX9-A76E (26 ng) (located in the dimerization domain (dim.)) had comparable activity, plotted as percentage of SOX9 WT. PQA, proline, glutamine and alanine-rich motif; PQS, proline, glutamine and serine-rich motif (Table 3). All experiments were conducted three times in duplicate. Error bars represent the standard error of mean values. Two-tail t-Test of paired sample means was performed. *** *P*<0.001, ** *P*<0.01, * *P*<0.05.

**Figure 5:**
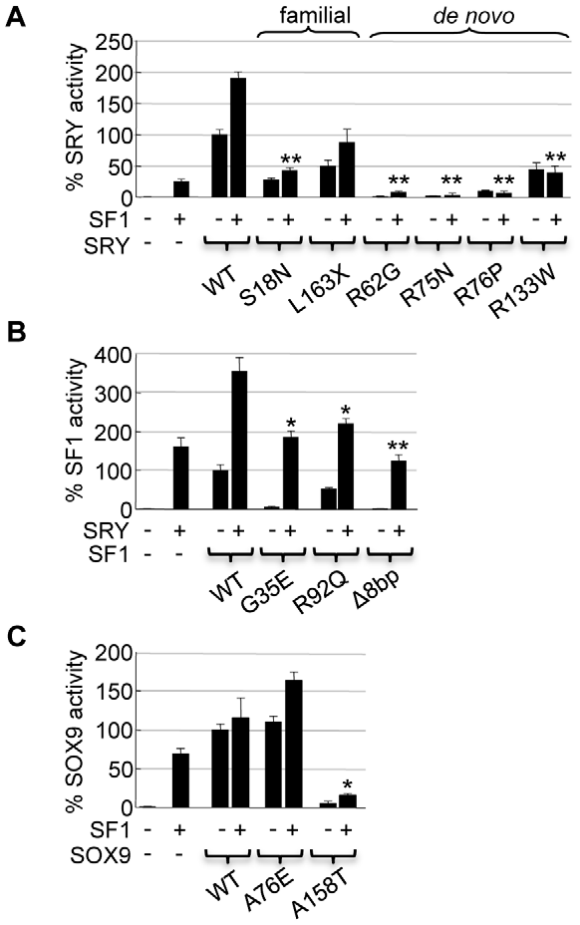
Mutant SRY, SF1 and SOX9 also fail to increase *SOX9* enhancer activity when in combination. (**A**) Co-transfection of SF1 expression plasmid with either SRY WT or SRY from 46,XY females demonstrates that *hTES* reporter activity, in the presence of SF1, is also significantly diminished. Statistical comparison made between SRY-WT/SF1 and SRY-Mutants/SF1. (**B**) Co-transfection with SRY and SF1 mutants also reveals significant reductions in *hTES* reporter activity compared to SRY and SF1 WT co-transfection. (**C**) In the presence of SF1, *hTES* reporter activity of the SOX9-A158T mutant is also significantly lower than that of SF1 together with SOX9 WT. All experiments were conducted at least twice with each transfection conducted in duplicate. Error bars represent the standard error of mean values. ** *P*<0.01, * *P*<0.05.

Patients with 46,XY gonadal dysgenesis carry mutations in testis-determining genes including *SRY*, *SF1* and *SOX9*. We used *hTES* as *in vitro* assay to study the impact of SRY, SF1 and SOX9 mutations on *SOX9* gene regulation. We first assayed the transcriptional activity of SRY from eight 46,XY females using the *hTES* enhancer. The SRY mutants varied in the location of SRY mutation, mode of inheritance (Fig. 4A) and biochemical defects (Table 1). Six of the eight SRY mutants tested showed a significant reduction in transcriptional activity (Fig. 4A). Of the *de novo* mutants with DNA binding defects, SRY-R62G, -R75N and -R76P showed ∼0–5% of wild-type SRY transcriptional activity, while the nuclear import mutant SRY-R133W showed ∼50% activity. Interestingly, the familial SRY-I90M mutant showed a significant ∼100% increase in *hTES* activation. This mutation is predicted to alter the hydrophobic isoleucine residue within a nuclear export sequence (Lx(1–3)Lx(2–3)LxL) suggesting correct SRY nuclear export could be important *in vivo*. Familial mutants inherited from fertile fathers (SRY-S18N, -R30I, -L163X) positioned outside of the HMG domain were investigated. Consistent with the partial penetrance of these mutations, their ability to activate *hTES* was consistently higher (∼50–90%) than that of the *de novo* mutants, highlighting the potency of this assay for analysis of mutations and the threshold level of activity needed for proper SRY function. In the presence of SF1, SRY mutants tested showed similar results with significant reductions in *hTES* stimulation (Fig. 5A).

**Table 1:** Summary of defects in SRY mutants.

			Mutant compared to wild type protein (%)			
SRY Mutants	Position	Phenotype	DNA Binding	DNA Bending	Nuclear Localization	*hTES* enhancer activation
S18N*	N terminal to HMG	PGD/CGD	∼90	∼100	ND	∼50
R30I*	N terminal to HMG	PGD/CGD	∼50	ND	ND	∼90
R62G	N-NLS	CGD	<1	∼67	∼25	<1
R75N	N-NLS	CGD	<1	ND	∼28	<1
R76P	N-NLS	CGD	∼33	∼95	∼50	∼5
I90M*	HMG	TH/PGD/CGD	∼95	∼100	ND	∼200
R133W	C-NLS	CGD	∼95	∼100	∼52	∼40
L163X*	C-terminal to HMG	CGD	ND	ND	ND	∼50

ND – Not determined.

*- denotes a familial mutation. N- and C- NLS – terminal nuclear localization signals. HMG – high mobility group domain. CGD – complete gonadal dysgenesis. PGD – partial gonadal dysgenesis. TH – true hermaphrodite. Biochemical defects obtained from respective references [27], [46], [51], [77].

*SF1* mutations cause 46,XY gonadal dysgenesis and adrenal defects in some cases [21], [23]. Three *SF1* mutations (SF1-G35E, -R92Q and -Δ8bp) localized to different regions of the SF1 protein affect DNA binding and transactivation of adrenal target genes [21], [39] (Table 2). We tested the ability of these SF1 mutants to activate *hTES* (Fig. 4B). The homozygous mutation SF1-R92Q showed ∼50% of wild-type transcriptional activity, consistent with the lack of phenotype in the heterozygous parents [21]. In contrast, heterozygous mutants SF1-G35E and SF1-Δ8bp showed no transcriptional activity. All three mutations significantly reduced the co-operative activation, with SRY, of *hTES* (Fig. 5B).

**Table 2:** Summary of defects in SF1 mutants.

				Mutant compared to wild type protein (%)		
SF1 Mutants	Position	Adrenal Failure	46,XY Gonadal Dysgenesis	DNA Binding	Transcriptional activity	*hTES* enhancer activation
G35E/WT	DBD	Yes	Yes	<1	<1	<5
R92Q/R92Q	FtzF1	Yes	Yes	∼50	∼25	∼50
Δ8bp/WT	LBD	No	Yes	∼50	∼30	<1

DBD – DNA binding domain. FtzF1 – FtzF1 box. LBD – Ligand binding domain. Biochemical defects obtained from respective references [21], [39]. Transcriptional activity for SF1 was assayed using reporter constructs for the *CYP11A*1 [21] and *CYP17*2 [39] adrenal genes respectively.

Heterozygous *SOX9* point mutations cause CD/SRA in most 46,XY individuals [2]. The SOX9 mutant, SOX9-A158T causes CD/SRA with 46,XY gonadal dysgenesis, due to defects in SOX9 nuclear import and DNA binding [40] (Table 3). The SOX9-A158T mutant failed to activate the *hTES* enhancer both alone (Fig. 4C) or in the presence of SF1 (Fig. 5C). In contrast, the SOX9-A76E mutant is encoded by a CD patient without 46,XY gonadal dysgenesis and lacks the ability to dimerize [41]. The SOX9-A76E mutant demonstrated wild-type activation of the *hTES* enhancer, consistent with dimerization of SOX9 not being required for sex determination.

**Table 3:** Summary of defects in SOX9 mutants.

				Mutant compared to wild type protein (%)		
SOX9 Mutants	Position	Phenotype	DNA Binding	DNA Bending	Nuclear Localization	*hTES* enhancer activation
A76E	Dimerization	CD alone	∼100	ND	∼100	∼100
A158T	HMG	CD with CGD	∼17	∼100	∼50	<1

HMG – high mobility group domain. CGD – complete gonadal dysgenesis. CD – Campomelic Dysplasia. Biochemical defects obtained from respective references [40], [41].

## Discussion

Regulation of *SOX9* expression in the gonad is tightly controlled. The key event that defines testis determination is the male-specific upregulation of *SOX9*. Results obtained in this study replicate the earliest developmental step in sex determination, the activation of *SOX9* transcription by SRY. Our findings have demonstrated a role for human SRY, SF1 and SOX9 in activating *SOX9* expression, endogenously in the NT2/D1 ‘Sertoli’-like cell line and also via a conserved human *SOX9* testis-specific enhancer, *hTES*. These same factors are involved in the initiation, upregulation and maintenance of Sox9 expression in XY gonads of mice [20].

Poor sequence conservation outside of the HMG domain between human and mouse SRY has led to the belief that human SRY may function through differing mechanisms. For example, mouse SRY has a transactivation domain that is absent in human SRY [26]. Furthermore, unlike the narrow window of transient expression of mouse Sry, beginning at E10.5 and ending abruptly at E12.5 [42], human SRY is first observed at E41 and is still present after 18 weeks gestation [43]. SRY function has been difficult to test due to the lack of *bone fide* target genes and good transcriptional assays. We demonstrate a role for SRY as a transcriptional activator, both on the *hTES* enhancer and on an endogenous target in NT2/D1 cells over-expressing SRY. In support of this, SRY was found to co-localise with transcriptionally active chromatin in NT2/D1 cells correlating with its proposed architectural property [27], but not pre-mRNA splicing as previously demonstrated [29]. Likewise, fusion of the transcriptional activator domain VP16 and not the repressor domain EnR to SRY resulted in increases in *hTES* reporter activity, suggesting that SRY is not merely repressing a repressor. While transcriptional activation of *SOX9* by SRY is clearly a pivotal process for proper testis development, the repressive actions of human SRY on ovary-determining pathways have also been proposed [44], [45].

The variability in transactivating properties of SRY, SF1 and SOX9 DSD mutants used in this study contribute to lower doses of SOX9 and provide important functional information. The transcriptional action of SRY identified here enabled us to investigate why certain *SRY* mutations always cause gonadal dysgenesis while other mutations do not. Certain sex-reversing SRY mutations show variable reduced DNA binding, DNA bending and nuclear import defects compared to wild type SRY (Table 1), however, studying the transcriptional activity of such mutants has been impossible due to a lack of known targets. Three missense *de novo* mutants, SRY-R62G, -R75N and -R76P located in the HMG box N-terminal NLS showed large losses in transcriptional activity of *hTES*. The *de novo* SRY-R133W mutant localised in the C-terminal NLS of SRY also showed a ∼50% loss in transcriptional activity in comparison to wild type SRY. These four mutants demonstrate the relative importance of DNA binding, bending and nuclear import for the activation of *hTES*. Of the four mutants, SRY-R62G and SRY-R75N have the most severe biochemical defects and this is also reflected by their transcriptional activation properties being the lowest (Table 1). The higher activation of *hTES* by SRY-R133W compared to SRY-R76P may be accounted for by its relatively normal DNA binding affinity. This result also reflects the importance of nuclear import to the proper function of SRY [46], [47]. SRY-R133W has ∼50% the nuclear import capacity of wild type SRY due to a loss in importin-β recognition [46]. Therefore, the use of such mutants in the assay developed in this study is somewhat more representative of SRY's action *in vivo*, where even a subtle mutation such as SRY-R133W may reduce the ability of SRY to up-regulate *SOX9*.

Intriguingly, the SRY-I90M mutation, which shows slightly reduced DNA binding and normal DNA bending by EMSA [27], activated the *hTES* enhancer ∼50% greater than that of wild type SRY. The location of this mutation at residue 90 coincides with a conserved hydrophobic residue present in a number of SOX proteins that forms part of a functional nuclear export signal (NES) [48]. Substitution of the hydrophobic amino acid position in the SOX9 NES (L142A) produces a protein unable to be exported from the nucleus. By analogy, the observed increase in transactivation of the SRY I90M mutant could be a consequence of increased nuclear accumulation. It is possible to speculate that a mutation that increases levels of nuclear SRY could be deleterious and the cause of 46,XY gonadal dysgenesis. This may also relate with the finding that an individual with a XYY karyotype, bearing two copies of the wildtype SRY coding sequence is female [49].

Patients with missense mutations lying outside of the HMG domain present as rare cases of 46,XY individuals with partial gonadal dysgenesis i.e. the two familial mutations SRY-S18N [50] and SRY-R30I [51]. The decrease in transcriptional activity of the SRY-S18N mutant points to a possible functional role for this N-terminal region. Interestingly, the same SRY-S18N mutation has also been isolated in a separate individual who presented with Turner syndrome and Y chromosome mosaicism (Ulrich Turner Syndrome) [52]. The SRY-R30I mutant was present in six 46,XY siblings where one patient showed complete gonadal dysgenesis, two showed partial gonadal dysgenesis and three were unaffected [51]. The SRY-R30I mutation is located near serine residues that when phosphorylated increase DNA binding [53]. SRY-R30I shows no loss in transcriptional activation of *hTES* and suggests a lesser importance on SRY phosphorylation. Together with the SRY-S18N mutant, it seems that in contrast to the severe biochemical defects of *de novo* mutations, inherited variants such as SRY-S18N and SRY-R30I produce SRY proteins with residual transactivating properties.

A third familial mutant tested was the nonsense mutation SRY-L163X that lacks the last 41 amino acids of the wild type SRY protein. This mutation showed a reduction in transcriptional activation of the *SOX9* enhancer, albeit moderate. The C-terminal region of SRY has two possible protein interacting domains, the PDZ and KRAB binding domains [54], [55]. The protein produced by this nonsense mutation still contains the KRAB binding region (amino acids 138–155, the bridge) but lacks the PDZ binding domain (the last 7 amino acids of the protein). The fact that the KRAB-O protein is theoretically still able to bind to this region and that recent work has demonstrated the potential role of KRAB-O in mouse sex determination [56], this does suggest that the first 163 amino acids of the SRY protein may be essential.

The incomplete penetrance of SRY mutants such as SRY-S18N, -R30I, -L163X together with the documentation of some families with fertile fathers being mosaic for both wild type and mutant SRY [57], [58], [59], does support the notion that dose dependency, genetic background and in extension, SOX9 activation levels, all play important roles in sex determination. With *Sry*, this can be demonstrated in mice where ectopic SRY activates *Sox9* in a dose-dependent manner [60]; expression of *Sry* transgene constructs below a critical threshold level in XX transgenic mice results in partial gonadal dysgenesis [33], [61], [62], [63], [64]; and *Sry* alleles from some mouse strains will cause gonadal dysgenesis when placed in certain genetic backgrounds [65], [66], [67], [68]. Whereas in humans the disruption to SF1 and SOX9 can result in 46,XY gonadal dysgenesis, haploinsufficiency of these critical factors in mice does not, making the assessment of essential SF1 or SOX9 dosage levels difficult in this context. In agreement to data obtained with the *mTES* [20], we have shown that both SF1 and SOX9 are also involved in the activation of *hTES*. In contrast to the *mTES*, our data does not reveal a synergistic activation between SF1 and SOX9. This result may reflect the sustained expression of human SRY in the XY gonad that is responsible, in addition to SF1 and SOX9, for maintaining *SOX9* levels. Indeed, human SRY is able to stimulate hTES on its own, whereas mouse SRY on *mTES* cannot [20].

Importantly, the SF1 and SOX9 transcriptional properties of mutants and levels of *hTES* activity can be related to DSD phenotypes. The SOX9 mutant, SOX9-A158T causes CD/SRA with 46,XY gonadal dysgenesis, due to defects in SOX9 nuclear import and DNA binding [40], whereas the SOX9-A76E mutant is encoded by a CD patient without 46,XY gonadal dysgenesis and lacks the ability to dimerize [41]. In fitting with previous observations, SOX9-A158T could not activate *hTES*, while the SOX9-A76E mutant demonstrated wild-type activation, consistent with dimerization of SOX9 not being required for sex determination. It is noteworthy that identical mutations in SOX9 can cause 46,XY DSD in one instance but not in another [69], [70]. While genetic background and SOX9 expression levels may be contributing to these diverse phenotypes, analysis of the transcriptional activities of such mutations in the context of the *hTES* assay warrant further investigation, as the activity of these mutants might be reduced to a threshold level.

The three SF1 mutants used all present 46,XY gonadal dysgenesis with SF1-G35E and SF1-R92Q mutants also showing adrenal failure (Table 2). The biochemical action of the SF1-G35E and SF1-R92Q mutants has previously been tested only on adrenal targets and not sex-determining genes [21], [71], [72]. Both SF1-G35E and SF1-R92Q on its own or in combination with SRY had reduced transcriptional activity on *hTES*, presumably through reduced DNA binding properties (Table 2). It is noteworthy that the homozygous SF1-R92Q mutant is recessive, familial in nature and that the gonadal dysgenesis phenotype only presents on certain genetic backgrounds [21]. The hypomorphic nature of this mutant reflects an ability to activate the *SOX9* enhancer to half the extent of SF1-WT. The SF1-Δ8bp mutant lacks half of the ligand binding domain and also the AF2 domain important for facilitating interactions with co-activators to stimulate transcription [73], although not sufficient alone [74]. We show a significant decrease in SF1-Δ8bp transcriptional activity on *hTES*. The SF1-Δ8bp has also been demonstrated to show a dominant-negative effect on wild type SF1 transcriptional activation of the *CYP17* promoter in human HEK293 (kidney), mouse MA10 (Leydig) and mouse Y1 (adrenal) cell lines [39]. The reduction in *hTES* activity, both alone and in co-operation with SRY, may therefore be a result of a dominant-negative effect of SF1-Δ8bp on endogenous SF1 or lack of co-factor recruitment in CHO cells, this warrants further investigation.

In conclusion, we have developed an *in vitro* assay that replicates the initial events of mammalian sex determination and puts into context the functional properties of mutations to SRY, SF1 and SOX9 in DSD.

## Materials and Methods

### Cell Culture and transfections

NT2/D1 (ATCC CRL-1973) cells were grown in Dulbecco's and Ham's F12 medium; CHO cells (ATCC CCL-61) were grown in Dulbecco's medium. Medium was supplemented with 10% fetal bovine serum and L-glutamine in an atmosphere of 5% CO_2_. Transient transfections were conducted using Fugene6 (Roche) in accordance with the manufacturer's instructions.

### Expression Plasmids

All mammalian expression plasmids were of pcDNA3 origin (Clontech) unless otherwise stated. DNA encoding wild-type Flag-tagged human SRY was previously described [46]. SRY mutants R62G, R75N, R76P and R133W were also previously described [46]. SRY mutants S18N, R30I, I90M and L163X were produced using the Site-directed Quick change mutagenesis kit (Qiagen) according to the manufacturer's instructions. pSlax-VP16 and -*Engrailed* shuttle vectors were a kind gift from Dr. Jonas Muhr as previously described [75] and were used to produce SRY fusion proteins. Briefly, the SRY ORF was amplified by PCR from pcDNA3-SRY using a forward oligonucleotide (containing *BamH*I, FLAG and KOZAK sequences) and a reverse oligonucleotide that replaces the stop codon with an *EcoR*I restriction site. The PCR product was then ligated to an *EcoR*I restriction site that was the beginning of the ORF of either VP16 or *Engrailed* domains. The fusion proteins were subsequently sub-cloned into pcDNA3. HA-tagged human SF1 and G35E SF1 mutant expression plasmids were kind gifts from Dr. Robert Viger as previously described [72]. Δ8bp SF1 mutant was a kind gift from Dr. Keith Parker as previously described [39]. The R92Q SF1 mutant was received as a gift from Dr. Larry Jameson as previously described [21]. pCI-neo-mSf1 (mouse cDNA) and the S203A phosphorylation mutant in the same expression plasmid were gifts from Dr. Holly Ingraham as previously described [38]. HA-tagged human SOX9 expression plasmid, SOX9-ΔPQA and SOX9-1-465 plasmids are previously described [76].

### Reporter Plasmids

The *hTES* sequence was amplified from the BAC contig clone RP11-84E24 (Genbank Accession AC007461), located on chromosome 17, by PCR using a forward oligonucleotide containing an *Xho*I restriction site (GATCATCCGCTCGAGCGGTGTTGAGAAGTGAACTGT) and a reverse oligonucleotide containing an *Acc*I restriction site (GATGGCCGGTCGACCGGCCACTTGGCTCAAATCTCAC). The resultant PCR product was cloned into the multiple cloning site of the E1b-CAT reporter construct [26]. Subsequently, the CAT reporter gene was replaced with the open reading frame of the *luciferase* reporter gene derived from the pGL3-basic (Promega). The *mTES* sequence contained within a shuttle vector was also sub-cloned into E1b-*luc*.

### Fluorescence activated cell sorting (FACS) and RNA extraction

NT2/D1 cells were seeded at a density of 2.5×10^5^ cells per well in 6-well plates 24 hours prior to transfection. In each transfection, pEF-GFP expression plasmid was added in a 1∶3 molar ratio with other expression plasmids to ensure efficient transfection efficiency. After 48 hours, cells were subjected to FACS. RNA was collected from GFP-positive cells using the RNeasy Mini Kit (Qiagen) in accordance with the manufacturer's instructions.

### Quantitative RT-PCR

0.5 µg of total RNA was reverse transcribed using the modifying enzyme Superscript III according to the manufacturer's specifications (Invitrogen). Real time quantification of mRNA levels was conducted using Lightcycler technology (Roche). Values obtained for each sample were standardized to amplification levels of the housekeeping gene *β-2-Microglobulin* (*β_2_M*). Standardized values were divided by vector alone transfectants to obtain total fold differences. Sequences of oligonucleotides used to amplify cDNA are (5′ to 3′ orientation): *SOX9* F-AAGACATTTAAGCTAAAGGCAACTCGTAC, R– TGATCACACGATTCTCCATCATCCTC; *β_2_M* F-TGAATTGCTATGTGTCTGGGT, R- CCTCCATGATGCTGCTTACAT.

### Reporter assays

CHO cells were seeded at a density of 2.3×10^5^ cells per well in 6-well plates 24 hours prior to transfection. 48 hours post-transfection the culture media was removed, cell lysate collected and *luciferase* or CAT reporter activity was measured according to the manufacturer's instructions (Promega). Reporter activity was normalized to *β-galactosidase* as an internal control (Promega). Empty reporter transfection data was divided from each transfection condition to standardize data. Fold activations were determined by dividing with vector alone transfection data.

### Immunohistochemistry

NT2/D1 cells used for immunohistochemistry were seeded on coverslips placed into 6-well plates. Standard protocols were used for immunohistochemistry. The primary antibodies used include affinity purified sheep anti-human SRY (1∶400), affinity purified rabbit anti-SOX9 (1∶400), affinity-purified mouse monoclonal anti-FLAG (1∶500) (Sigma), mouse Anti-SC35 monoclonal antibody (1∶1000) (Sigma) and the rabbit Anti-Tri-methylated lysine 4 of histone 3 (H3 Me_3_K4) polyclonal antibody (1∶200) (Upstate Biotech). The secondary antibodies used include Alexa 488-conjugated donkey anti-rabbit IgG (1∶800), Alexa 598-conjugated donkey anti-mouse IgG (1∶800) (Molecular Probes). Coverslips were mounted onto slides with DAKO fluorescence mounting medium containing DAPI (final 0.6 mg/ml). Images were captured using an Olympus FV500 confocal laser scanning microscope. Image analysis was performed using NIH ImageJ (public domain software).

### *In vitro* translation and EMSA analysis of SRY fusion proteins

Proteins were produced using an *in vitro* coupled transcription and translation process using a TNT Coupled Reticulocyte Lysate System (Promega) according to the manufacturer's guidelines. A small proportion of each sample was translated with the incorporation of ^35^S-Methionine to produce a radio-labeled protein to enable verification that the protein produced was of the correct molecular mass. EMSA analysis was performed using *in vitro* translated protein combined with 1 µl of ^32^P-labeled DNA probe (∼40,000 cpm) using standard protocols. Oligonucleotide sequence of the SRY consensus probe was (5′ to 3′ orientation) GGGTTAACTAAACAATAGAATCTGGTAGA; core binding sequence is underlined. The gel was visualized using the Storm phosphor-imaging system and ImageQuant analysis software (Amersham).

## References

[1] Hughes IA, Houk C, Ahmed SF, Lee PA (2006). Consensus statement on management of intersex disorders.. J Pediatr Urol.

[2] Cameron FJ, Sinclair AH (1997). Mutations in SRY and SOX9: testis-determining genes.. Hum Mutat.

[3] McElreavey K, Fellous M (1999). Sex determination and the Y chromosome.. Am J Med Genet.

[4] Sekido R, Lovell-Badge R (2009). Sex determination and SRY: down to a wink and a nudge?. Trends Genet.

[5] Rossi P, Dolci S, Albanesi C, Grimaldi P, Geremia R (1993). Direct evidence that the mouse sex-determining gene Sry is expressed in the somatic cells of male fetal gonads and in the germ cell line in the adult testis.. Mol Reprod Dev.

[6] Capel B, Albrecht KH, Washburn LL, Eicher EM (1999). Migration of mesonephric cells into the mammalian gonad depends on Sry.. Mech Dev.

[7] Schmahl J, Eicher EM, Washburn LL, Capel B (2000). Sry induces cell proliferation in the mouse gonad.. Development.

[8] Brennan J, Karl J, Capel B (2002). Divergent vascular mechanisms downstream of Sry establish the arterial system in the XY gonad.. Dev Biol.

[9] Matoba S, Kanai Y, Kidokoro T, Kanai-Azuma M, Kawakami H (2005). A novel Sry-downstream cellular event which preserves the readily available energy source of glycogen in mouse sex differentiation.. J Cell Sci.

[10] Foster JW, Dominguez-Steglich MA, Guioli S, Kowk G, Weller PA (1994). Campomelic dysplasia and autosomal sex reversal caused by mutations in an SRY-related gene.. Nature.

[11] Wagner T, Wirth J, Meyer J, Zabel B, Held M (1994). Autosomal sex reversal and campomelic dysplasia are caused by mutations in and around the SRY-related gene SOX9.. Cell.

[12] Just W, Rau W, Vogel W, Akhverdian M, Fredga K (1995). Absence of Sry in species of the vole Ellobius.. Nat Genet.

[13] Oreal E, Pieau C, Mattei MG, Josso N, Picard JY (1998). Early expression of AMH in chicken embryonic gonads precedes testicular SOX9 expression.. Dev Dyn.

[14] Moreno-Mendoza N, Harley VR, Merchant-Larios H (1999). Differential expression of SOX9 in gonads of the sea turtle Lepidochelys olivacea at male- or female-promoting temperatures.. J Exp Zool.

[15] Western PS, Harry JL, Graves JA, Sinclair AH (1999). Temperature-dependent sex determination: upregulation of SOX9 expression after commitment to male development.. Dev Dyn.

[16] Huang B, Wang S, Ning Y, Lamb AN, Bartley J (1999). Autosomal XX sex reversal caused by duplication of SOX9.. Am J Med Genet.

[17] Bishop CE, Whitworth DJ, Qin Y, Agoulnik AI, Agoulnik IU (2000). A transgenic insertion upstream of sox9 is associated with dominant XX sex reversal in the mouse.. Nat Genet.

[18] Vidal VP, Chaboissier MC, de Rooij DG, Schedl A (2001). Sox9 induces testis development in XX transgenic mice.. Nat Genet.

[19] Refai O, Friedman A, Terry L, Jewett T, Pearlman A (2010). De novo 12;17 translocation upstream of SOX9 resulting in 46,XX testicular disorder of sex development.. Am J Med Genet A.

[20] Sekido R, Lovell-Badge R (2008). Sex determination involves synergistic action of SRY and SF1 on a specific Sox9 enhancer.. Nature.

[21] Achermann JC, Ozisik G, Ito M, Orun UA, Harmanci K (2002). Gonadal determination and adrenal development are regulated by the orphan nuclear receptor steroidogenic factor-1, in a dose-dependent manner.. J Clin Endocrinol Metab.

[22] Lin L, Philibert P, Ferraz-de-Souza B, Kelberman D, Homfray T (2007). Heterozygous missense mutations in steroidogenic factor 1 (SF1/Ad4BP, NR5A1) are associated with 46,XY disorders of sex development with normal adrenal function.. J Clin Endocrinol Metab.

[23] Kohler B, Lin L, Ferraz-de-Souza B, Wieacker P, Heidemann P (2008). Five novel mutations in steroidogenic factor 1 (SF1, NR5A1) in 46,XY patients with severe underandrogenization but without adrenal insufficiency.. Hum Mutat.

[24] Whitfield LS, Lovell-Badge R, Goodfellow PN (1993). Rapid sequence evolution of the mammalian sex-determining gene SRY.. Nature.

[25] Lovell-Badge R, Canning C, Sekido R (2002). Sex-determining genes in mice: building pathways.. Novartis Found Symp.

[26] Dubin RA, Ostrer H (1994). Sry is a transcriptional activator.. Mol Endocrinol.

[27] Pontiggia A, Rimini R, Harley VR, Goodfellow PN, Lovell-Badge R (1994). Sex-reversing mutations affect the architecture of SRY-DNA complexes.. Embo J.

[28] McElreavey K, Vilain E, Abbas N, Herskowitz I, Fellous M (1993). A regulatory cascade hypothesis for mammalian sex determination: SRY represses a negative regulator of male development.. Proc Natl Acad Sci U S A.

[29] Ohe K, Lalli E, Sassone-Corsi P (2002). A direct role of SRY and SOX proteins in pre-mRNA splicing.. Proc Natl Acad Sci U S A.

[30] Knower KC, Sim H, McClive PJ, Bowles J, Koopman P (2007). Characterisation of urogenital ridge gene expression in the human embryonal carcinoma cell line NT2/D1.. Sex Dev.

[31] Ferrari S, Harley VR, Pontiggia A, Goodfellow PN, Lovell-Badge R (1992). SRY, like HMG1, recognizes sharp angles in DNA.. Embo J.

[32] Santos-Rosa H, Schneider R, Bannister AJ, Sherriff J, Bernstein BE (2002). Active genes are tri-methylated at K4 of histone H3.. Nature.

[33] Sekido R, Bar I, Narvaez V, Penny G, Lovell-Badge R (2004). SOX9 is up-regulated by the transient expression of SRY specifically in Sertoli cell precursors.. Dev Biol.

[34] Bagheri-Fam S, Sinclair AH, Koopman P, Harley VR (2010). Conserved regulatory modules in the Sox9 testis-specific enhancer predict roles for SOX, TCF/LEF, Forkhead, DMRT, and GATA proteins in vertebrate sex determination.. Int J Biochem Cell Biol.

[35] Cohen DR, Sinclair AH, McGovern JD (1994). SRY protein enhances transcription of Fos-related antigen 1 promoter constructs.. Proc Natl Acad Sci U S A.

[36] Friedman AD, Triezenberg SJ, McKnight SL (1988). Expression of a truncated viral trans-activator selectively impedes lytic infection by its cognate virus.. Nature.

[37] Han K, Manley JL (1993). Functional domains of the Drosophila Engrailed protein.. Embo J.

[38] Hammer GD, Krylova I, Zhang Y, Darimont BD, Simpson K (1999). Phosphorylation of the nuclear receptor SF-1 modulates cofactor recruitment: integration of hormone signaling in reproduction and stress.. Mol Cell.

[39] Correa RV, Domenice S, Bingham NC, Billerbeck AE, Rainey WE (2004). A microdeletion in the ligand binding domain of human steroidogenic factor 1 causes XY sex reversal without adrenal insufficiency.. J Clin Endocrinol Metab.

[40] Preiss S, Argentaro A, Clayton A, John A, Jans DA (2001). Compound effects of point mutations causing campomelic dysplasia/autosomal sex reversal upon SOX9 structure, nuclear transport, DNA binding, and transcriptional activation.. J Biol Chem.

[41] Bernard P, Tang P, Liu S, Dewing P, Harley V (2003). Dimerization of SOX9 is required for chondrogenesis, but not for sex determination.. Hum Mol Genet.

[42] Hacker A, Capel B, Goodfellow P, Lovell-Badge R (1995). Expression of Sry, the mouse sex determining gene.. Development.

[43] Hanley NA, Hagan DM, Clement-Jones M, Ball SG, Strachan T (2000). SRY, SOX9, and DAX1 expression patterns during human sex determination and gonadal development.. Mech Dev.

[44] Bernard P, Sim H, Knower K, Vilain E, Harley V (2008). Human SRY inhibits beta-catenin-mediated transcription.. Int J Biochem Cell Biol.

[45] Lau YF, Li Y (2009). The human and mouse sex-determining SRY genes repress the Rspol/beta-catenin signaling.. J Genet Genomics.

[46] Harley VR, Layfield S, Mitchell CL, Forwood JK, John AP (2003). Defective importin {beta} recognition and nuclear import of the sex-determining factor SRY are associated with XY sex-reversing mutations.. Proc Natl Acad Sci U S A.

[47] Li B, Zhang W, Chan G, Jancso-Radek A, Liu S (2001). Human sex reversal due to impaired nuclear localization of SRY: a clinical correlation.. J Biol Chem.

[48] Gasca S, Canizares J, De Santa Barbara P, Mejean C, Poulat F (2002). A nuclear export signal within the high mobility group domain regulates the nucleocytoplasmic translocation of SOX9 during sexual determination.. Proc Natl Acad Sci U S A.

[49] Benasayag S, Rittler M, Nieto F, Torres de Aguirre N, Reyes M (2001). 47,XYY karyotype and normal SRY in a patient with a female phenotype.. J Pediatr Endocrinol Metab.

[50] Domenice S, Yumie Nishi M, Correia Billerbeck AE, Latronico AC, Aparecida Medeiros M (1998). A novel missense mutation (S18N) in the 5′ non-HMG box region of the SRY gene in a patient with partial gonadal dysgenesis and his normal male relatives.. Hum Genet.

[51] Assumpcao JG, Benedetti CE, Maciel-Guerra AT, Guerra G, Baptista MT (2002). Novel mutations affecting SRY DNA-binding activity: the HMG box N65H associated with 46,XY pure gonadal dysgenesis and the familial non-HMG box R30I associated with variable phenotypes.. J Mol Med.

[52] Canto P, de la Chesnaye E, Lopez M, Cervantes A, Chavez B (2000). A mutation in the 5′ non-high mobility group box region of the SRY gene in patients with Turner syndrome and Y mosaicism.. J Clin Endocrinol Metab.

[53] Desclozeaux M, Poulat F, de Santa Barbara P, Capony JP, Turowski P (1998). Phosphorylation of an N-terminal motif enhances DNA-binding activity of the human SRY protein.. J Biol Chem.

[54] Poulat F, Barbara PS, Desclozeaux M, Soullier S, Moniot B (1997). The human testis determining factor SRY binds a nuclear factor containing PDZ protein interaction domains.. J Biol Chem.

[55] Oh HJ, Li Y, Lau YF (2005). Sry associates with the heterochromatin protein 1 complex by interacting with a KRAB domain protein.. Biol Reprod.

[56] Polanco JC, Wilhelm D, Mizusaki H, Jackson A, Browne C (2009). Functional analysis of the SRY-KRAB interaction in mouse sex determination.. Biol Cell.

[57] Barbosa AS, Ferraz-Costa TE, Semer M, Liberman B, Moreira-Filho CA (1995). XY gonadal dysgenesis and gonadoblastoma: a study in two sisters with a cryptic deletion of the Y chromosome involving the SRY gene.. Hum Genet.

[58] Bilbao JR, Loridan L, Castano L (1996). A novel postzygotic nonsense mutation in SRY in familial XY gonadal dysgenesis.. Hum Genet.

[59] Hines RS, Tho SP, Zhang YY, Plouffe L, Hansen KA (1997). Paternal somatic and germ-line mosaicism for a sex-determining region on Y (SRY) missense mutation leading to recurrent 46,XY sex reversal.. Fertil Steril.

[60] Kidokoro T, Matoba S, Hiramatsu R, Fujisawa M, Kanai-Azuma M (2005). Influence on spatiotemporal patterns of a male-specific Sox9 activation by ectopic Sry expression during early phases of testis differentiation in mice.. Dev Biol.

[61] Capel B, Rasberry C, Dyson J, Bishop CE, Simpson E (1993). Deletion of Y chromosome sequences located outside the testis determining region can cause XY female sex reversal.. Nat Genet.

[62] Lovell-Badge R (1993). Sex determining gene expression during embryogenesis.. Philos Trans R Soc Lond B Biol Sci.

[63] Swain A, Narvaez V, Burgoyne P, Camerino G, Lovell-Badge R (1998). Dax1 antagonizes Sry action in mammalian sex determination.. Nature.

[64] Burgoyne PS, Lovell-Badge R, Rattigan A (2001). Evidence that the testis determination pathway interacts with a non-dosage compensated, X-linked gene.. Int J Dev Biol.

[65] Eicher EM, Washburn LL, Schork NJ, Lee BK, Shown EP (1996). Sex-determining genes on mouse autosomes identified by linkage analysis of C57BL/6J-YPOS sex reversal.. Nat Genet.

[66] Lee CH, Taketo T (2001). Low levels of Sry transcripts cannot be the sole cause of B6-Y(TIR) sex reversal.. Genesis.

[67] Albrecht KH, Young M, Washburn LL, Eicher EM (2003). Sry Expression Level and Protein Isoform Differences Play a Role in Abnormal Testis Development in C57BL/6J Mice Carrying Certain Sry Alleles.. Genetics.

[68] Bullejos M, Koopman P (2005). Delayed Sry and Sox9 expression in developing mouse gonads underlies B6-Y(DOM) sex reversal.. Dev Biol.

[69] Kwok C, Weller PA, Guioli S, Foster JW, Mansour S (1995). Mutations in SOX9, the gene responsible for Campomelic dysplasia and autosomal sex reversal.. Am J Hum Genet.

[70] Hageman RM, Cameron FJ, Sinclair AH (1998). Mutation analysis of the SOX9 gene in a patient with campomelic dysplasia.. Hum Mutat Suppl.

[71] Ito M, Achermann JC, Jameson JL (2000). A naturally occurring steroidogenic factor-1 mutation exhibits differential binding and activation of target genes.. J Biol Chem.

[72] Tremblay JJ, Viger RS (2003). A mutated form of steroidogenic factor 1 (SF-1 G35E) that causes sex reversal in humans fails to synergize with transcription factor GATA-4.. J Biol Chem.

[73] Steinmetz AC, Renaud JP, Moras D (2001). Binding of ligands and activation of transcription by nuclear receptors.. Annu Rev Biophys Biomol Struct.

[74] Crawford PA, Polish JA, Ganpule G, Sadovsky Y (1997). The activation function-2 hexamer of steroidogenic factor-1 is required, but not sufficient for potentiation by SRC-1.. Mol Endocrinol.

[75] Sandberg M, Kallstrom M, Muhr J (2005). Sox21 promotes the progression of vertebrate neurogenesis.. Nat Neurosci.

[76] McDowall S, Argentaro A, Ranganathan S, Weller P, Mertin S (1999). Functional and structural studies of wild type SOX9 and mutations causing campomelic dysplasia.. J Biol Chem.

[77] Mitchell CL, Harley VR (2002). Biochemical defects in eight SRY missense mutations causing XY gonadal dysgenesis.. Mol Genet Metab.

